# Supplementary figures and images for: The Effect of CDKN1A on the Expression of Genes Related to Milk Protein and Milk Fat Synthesis in Bovine Mammary Epithelial Cells
Source: Vet Sci. 2025 Jun 1;12(6):534. doi: 10.3390/vetsci12060534 (PMC12197750; doi:10.3390/vetsci12060534)

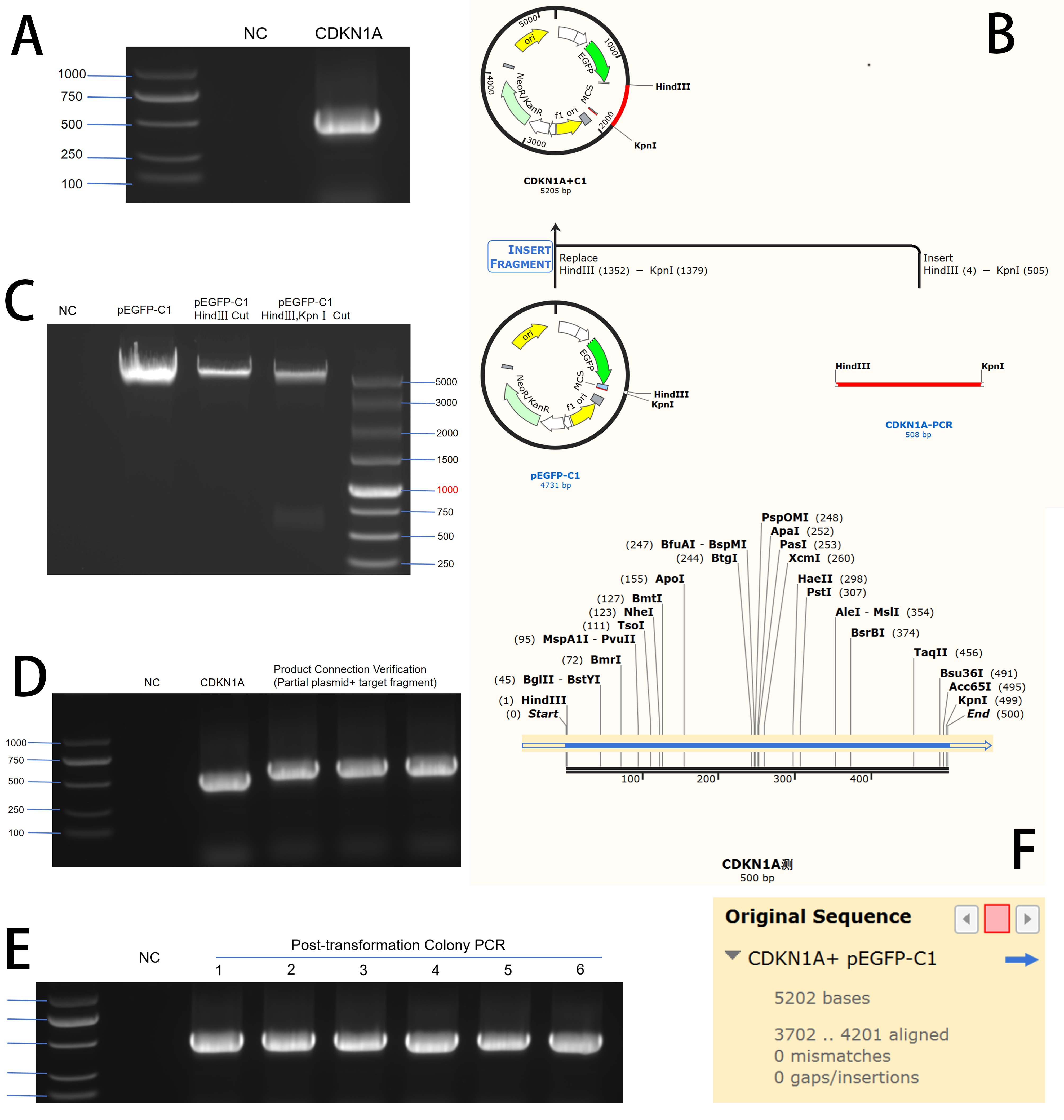

Supplement: Supplementary file 1 [file vetsci-12-00534-s001.zip › Original images/PCR/Fig. 1.png]

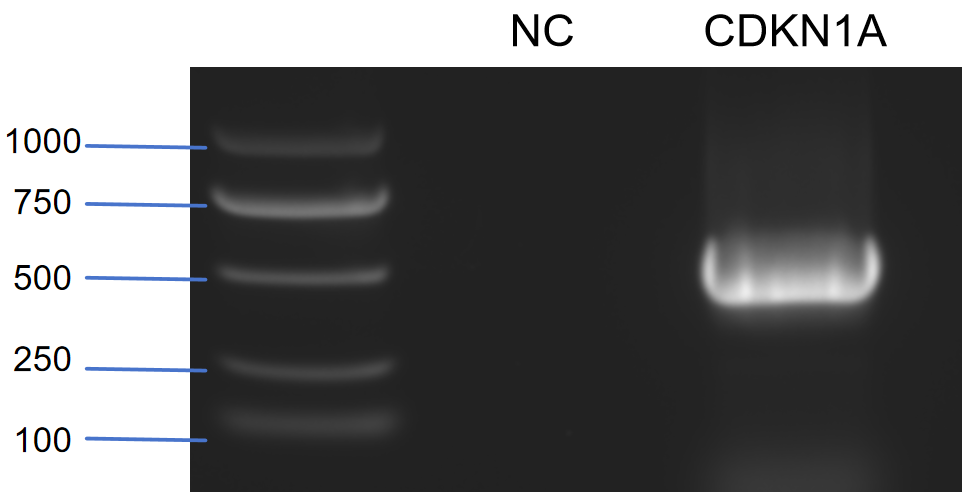

Supplement: Supplementary file 1 [file vetsci-12-00534-s001.zip › Original images/PCR/Fig.1-A.png]

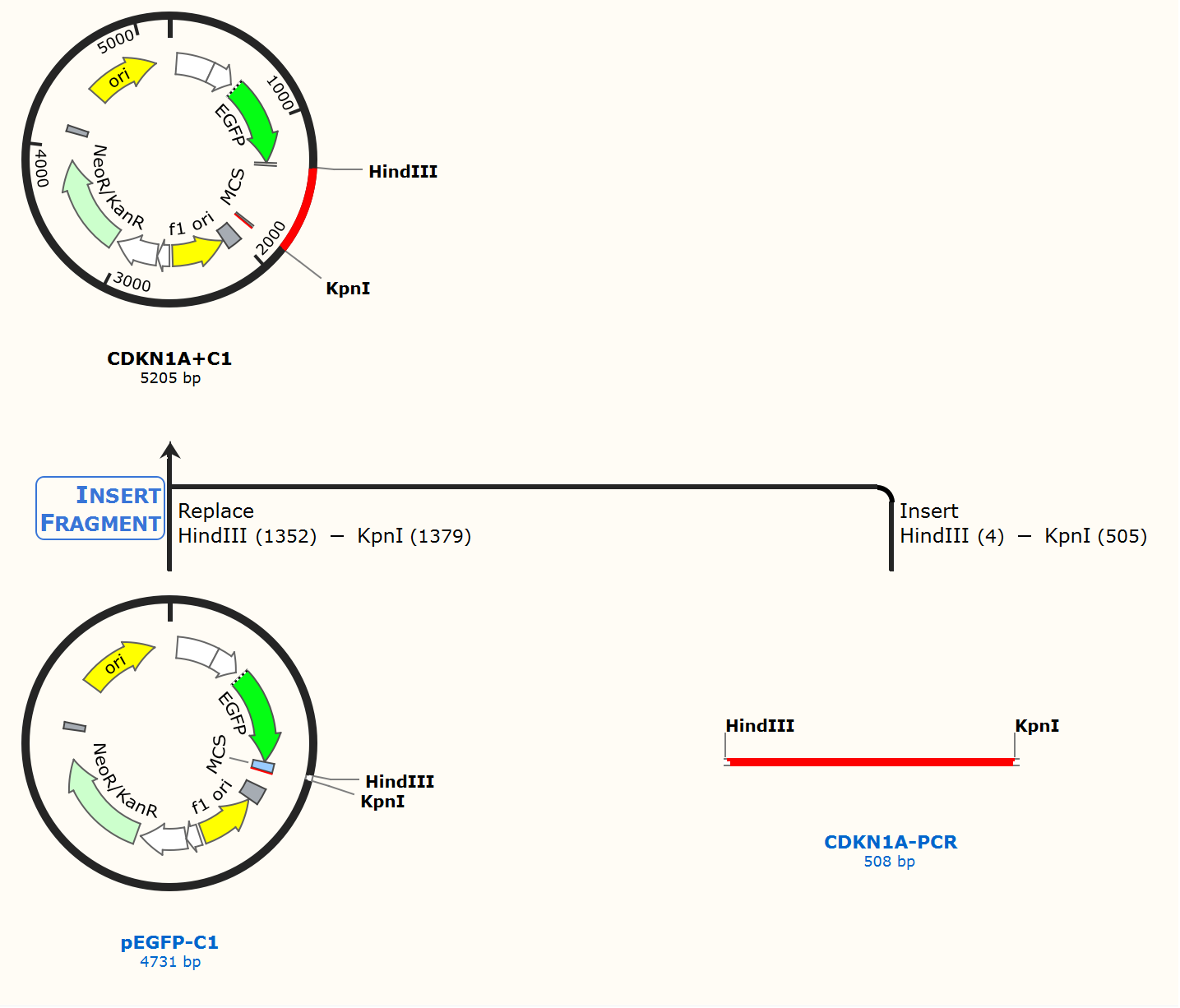

Supplement: Supplementary file 1 [file vetsci-12-00534-s001.zip › Original images/PCR/Fig.1-B.png]

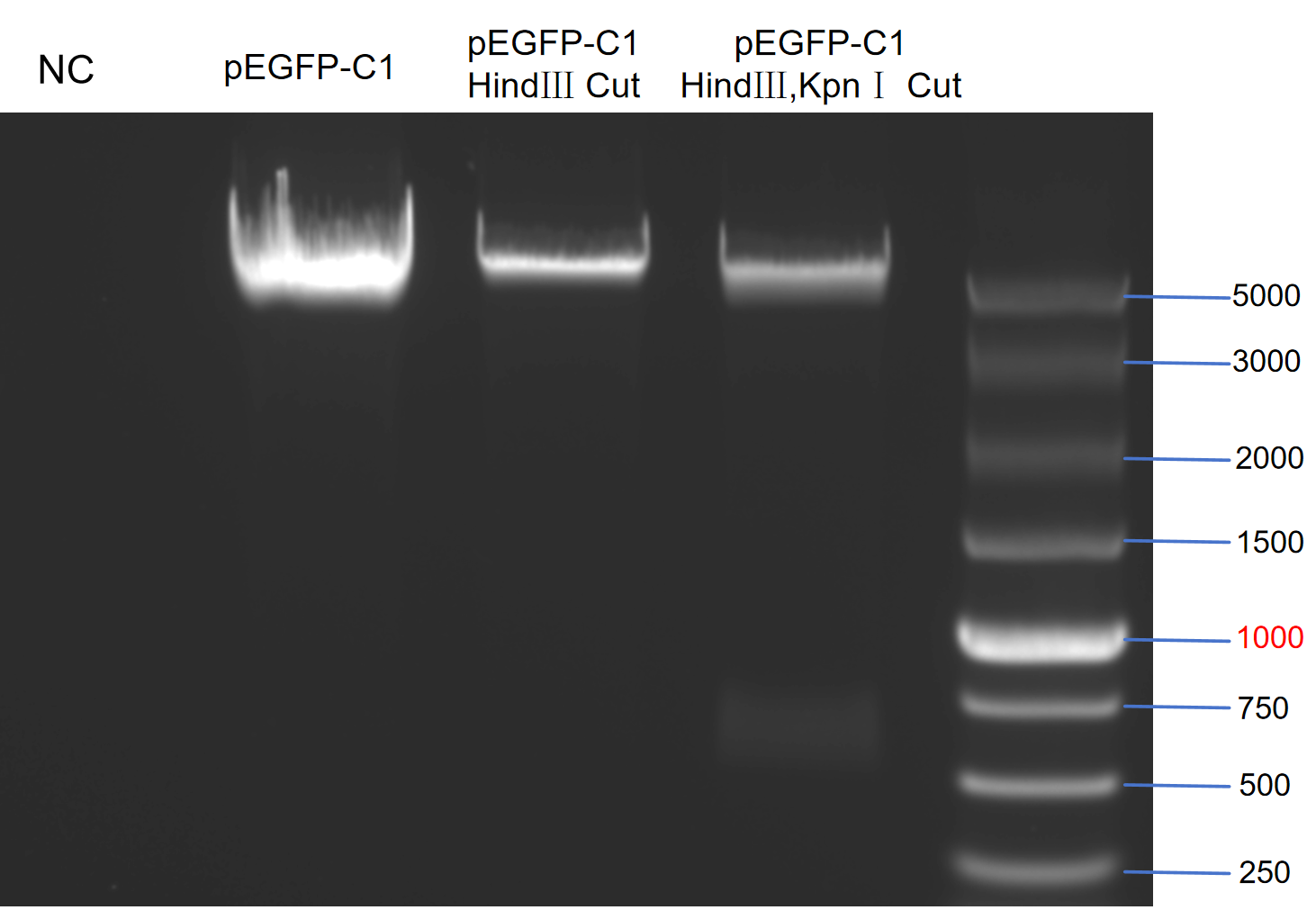

Supplement: Supplementary file 1 [file vetsci-12-00534-s001.zip › Original images/PCR/Fig.1-C.png]

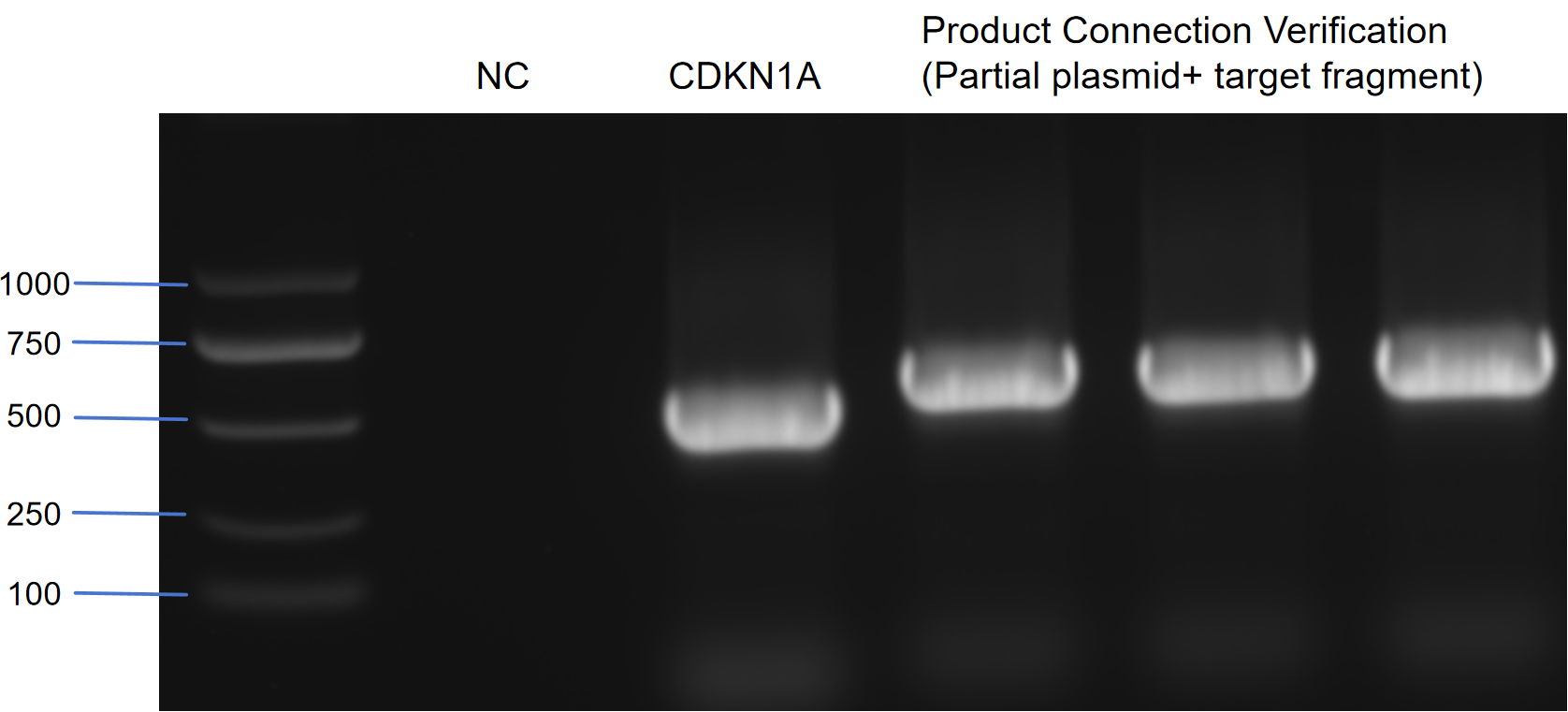

Supplement: Supplementary file 1 [file vetsci-12-00534-s001.zip › Original images/PCR/Fig.1-D.png]

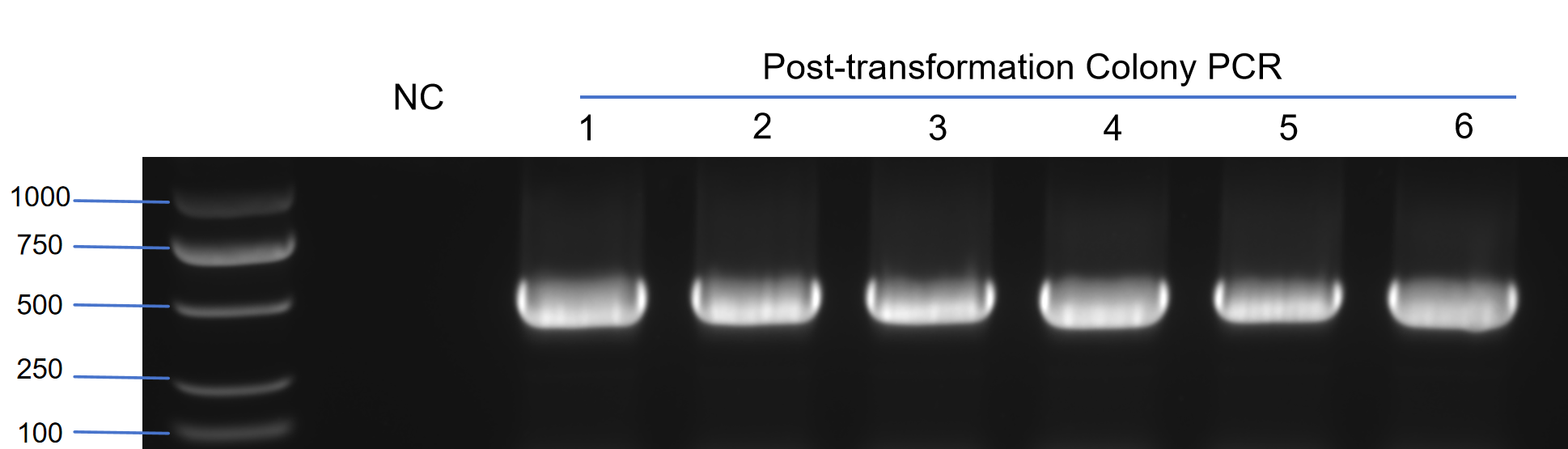

Supplement: Supplementary file 1 [file vetsci-12-00534-s001.zip › Original images/PCR/Fig.1-E.png]

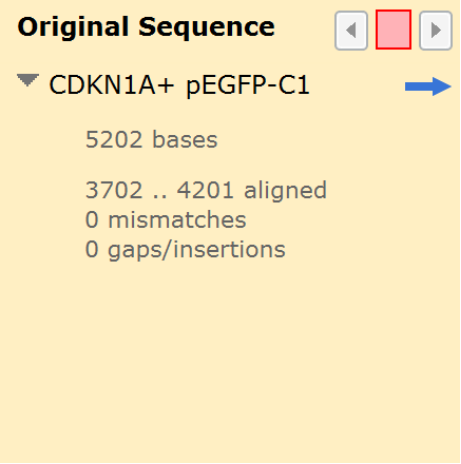

Supplement: Supplementary file 1 [file vetsci-12-00534-s001.zip › Original images/PCR/Fig.1-F1.png]

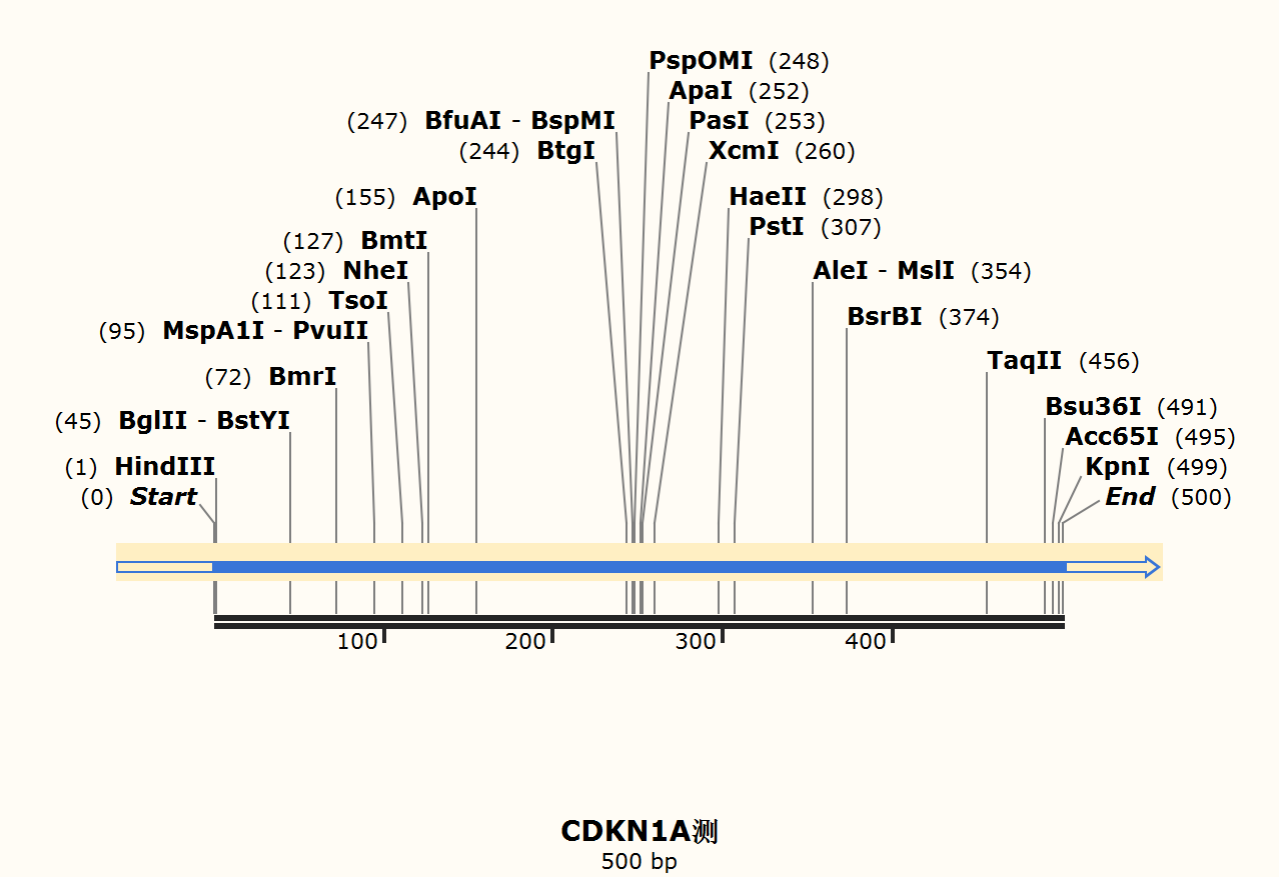

Supplement: Supplementary file 1 [file vetsci-12-00534-s001.zip › Original images/PCR/Fig.1-F2.png]

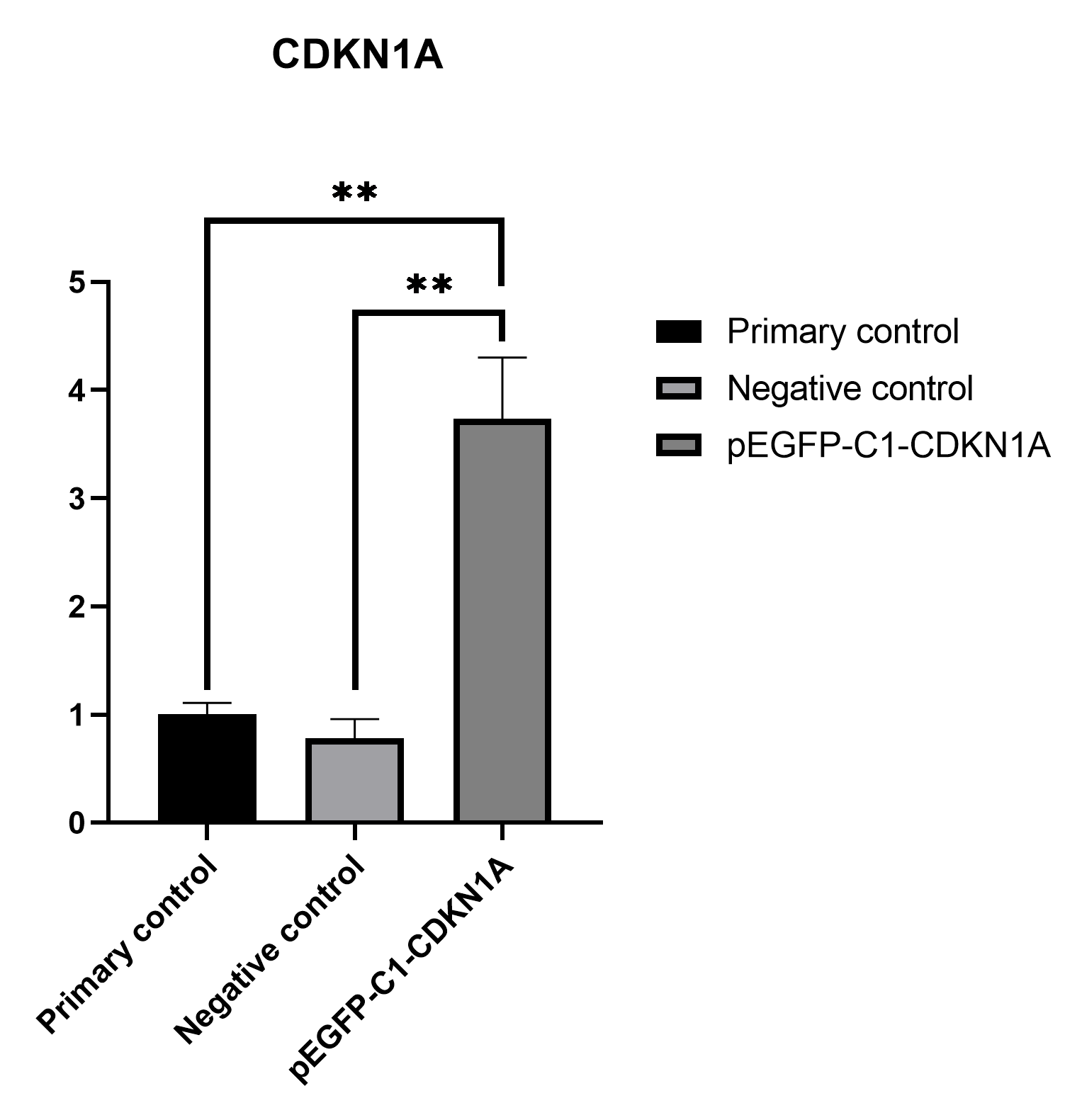

Supplement: Supplementary file 1 [file vetsci-12-00534-s001.zip › Original images/QPCR/Fig. 2-A.tif]

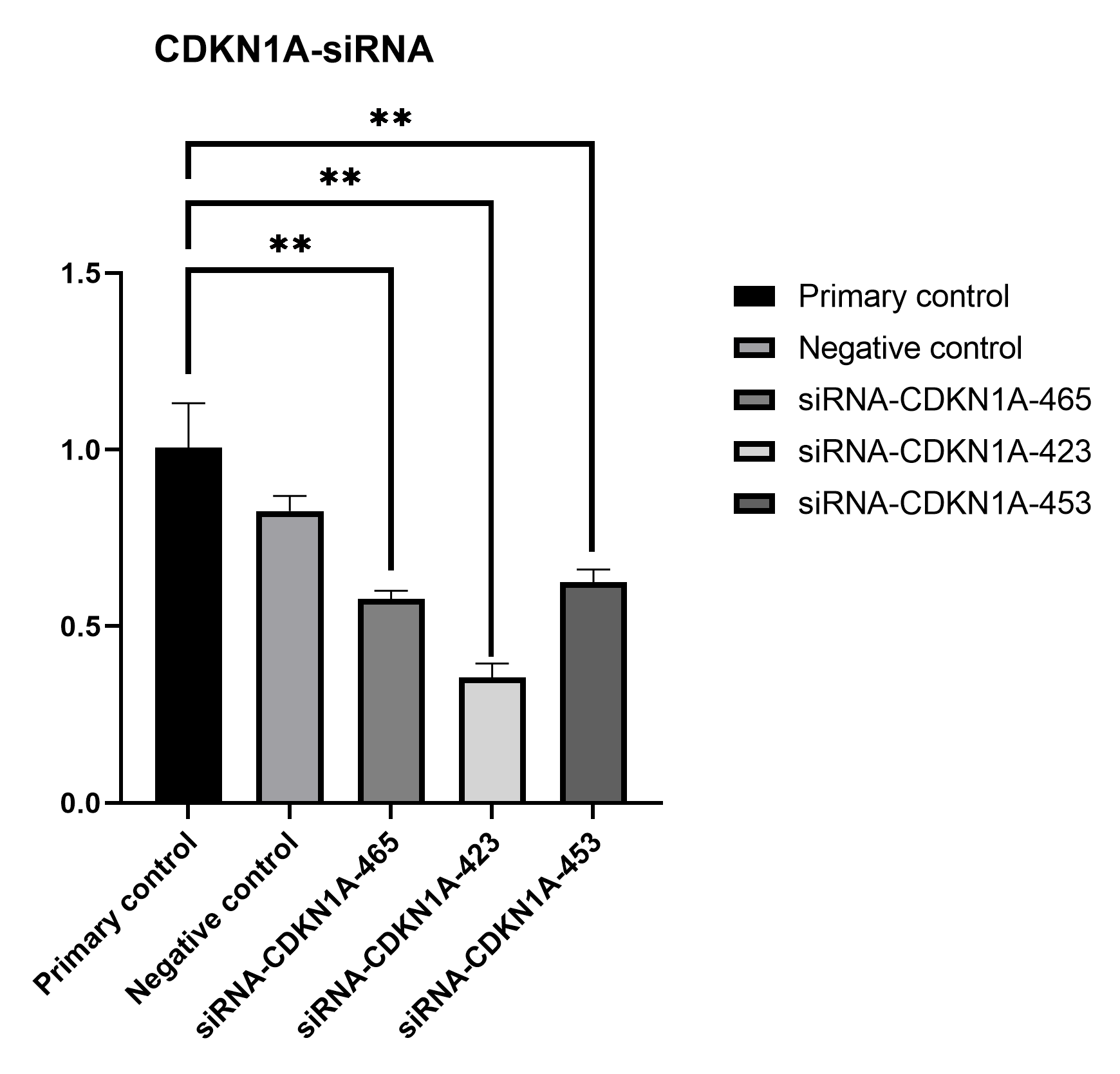

Supplement: Supplementary file 1 [file vetsci-12-00534-s001.zip › Original images/QPCR/Fig. 2-B.tif]

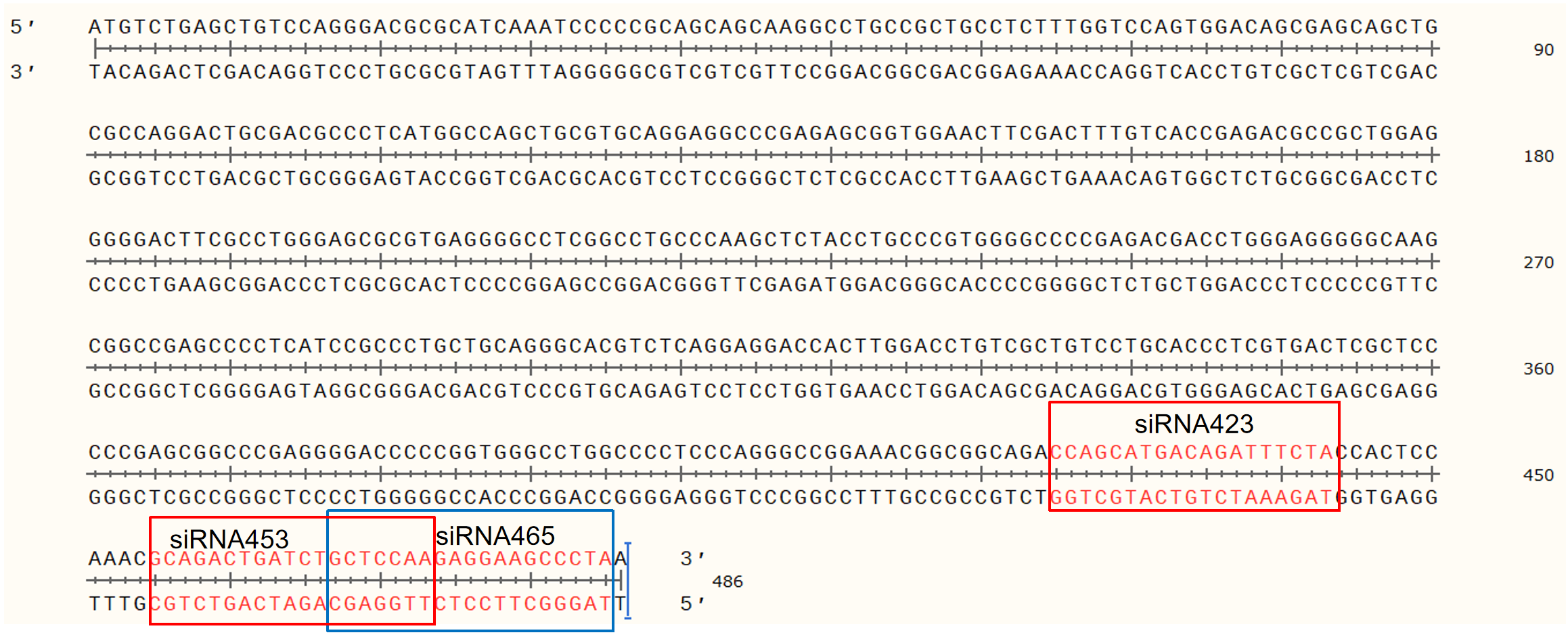

Supplement: Supplementary file 1 [file vetsci-12-00534-s001.zip › Original images/QPCR/Fig. 2-C.png]

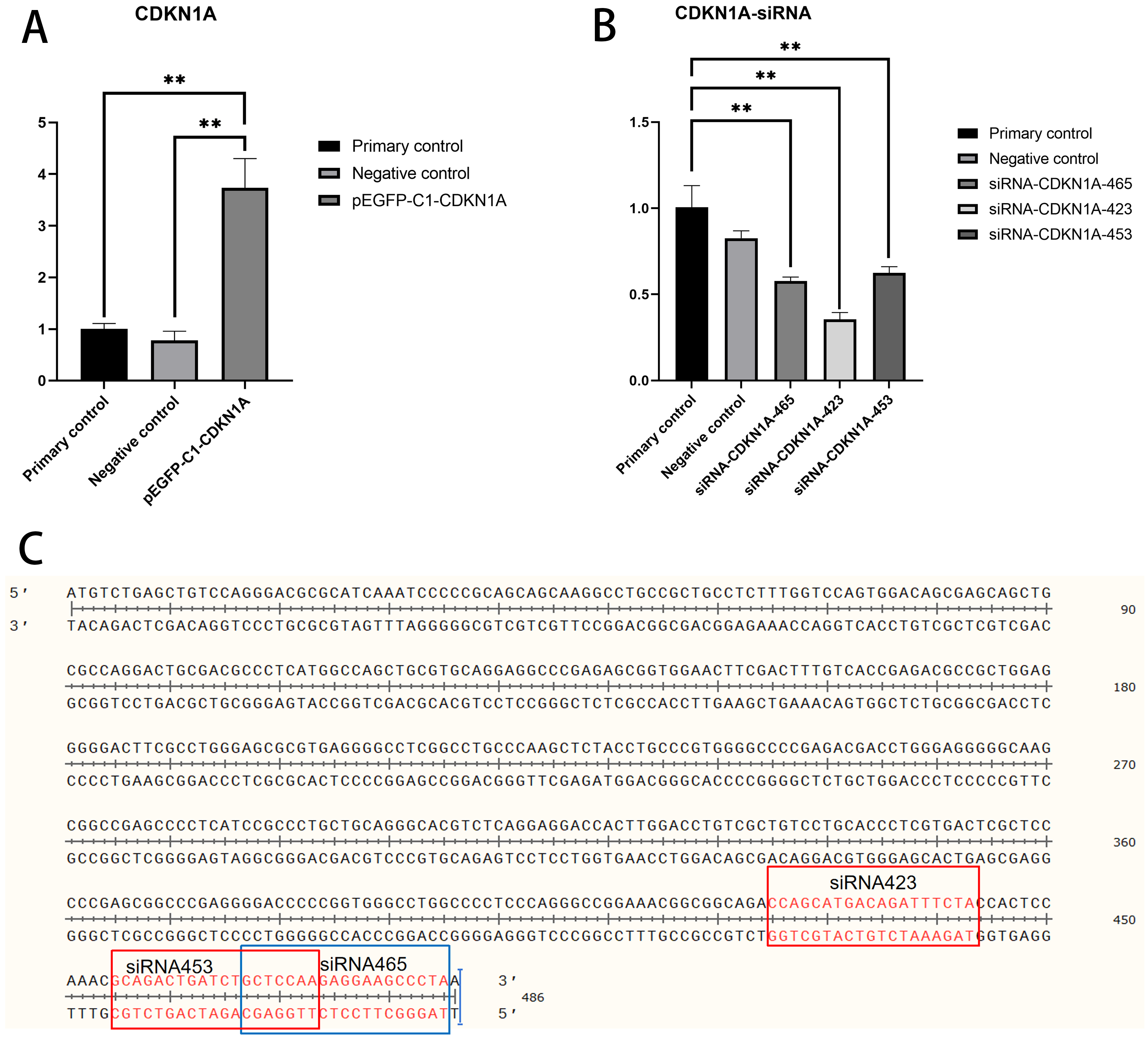

Supplement: Supplementary file 1 [file vetsci-12-00534-s001.zip › Original images/QPCR/Fig. 2.png]

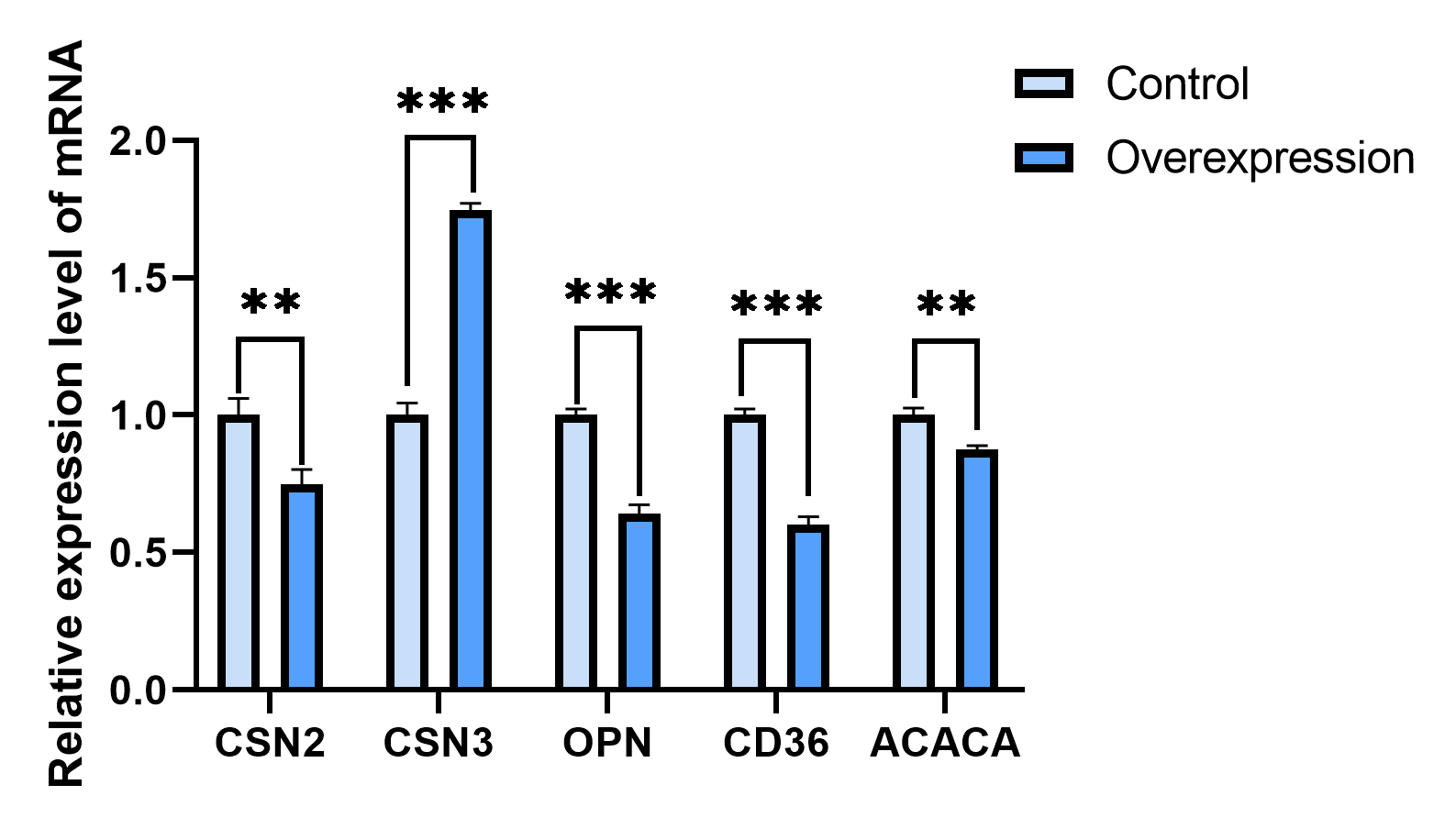

Supplement: Supplementary file 1 [file vetsci-12-00534-s001.zip › Original images/QPCR/Fig. 3-A.tif]

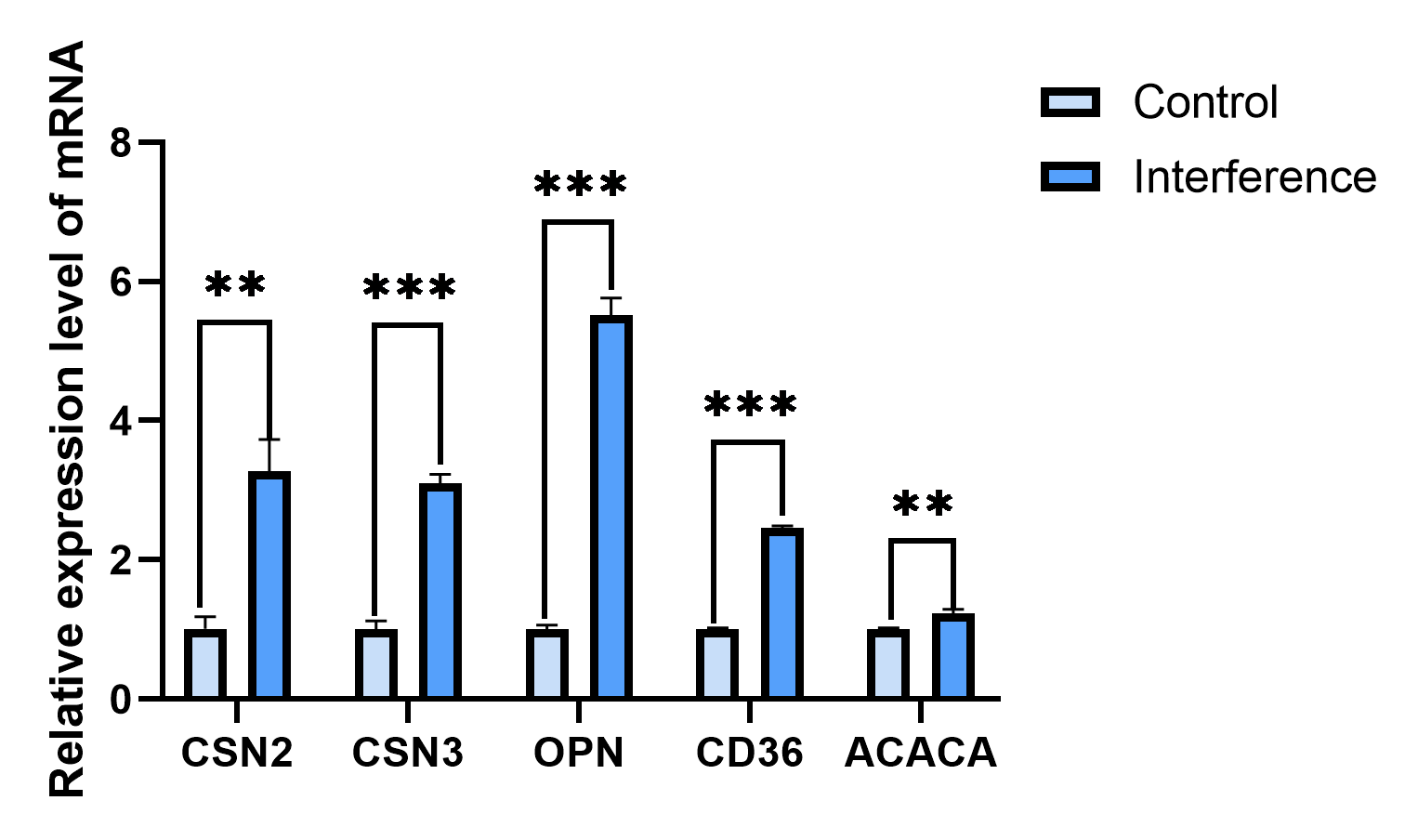

Supplement: Supplementary file 1 [file vetsci-12-00534-s001.zip › Original images/QPCR/Fig. 3-B.tif]

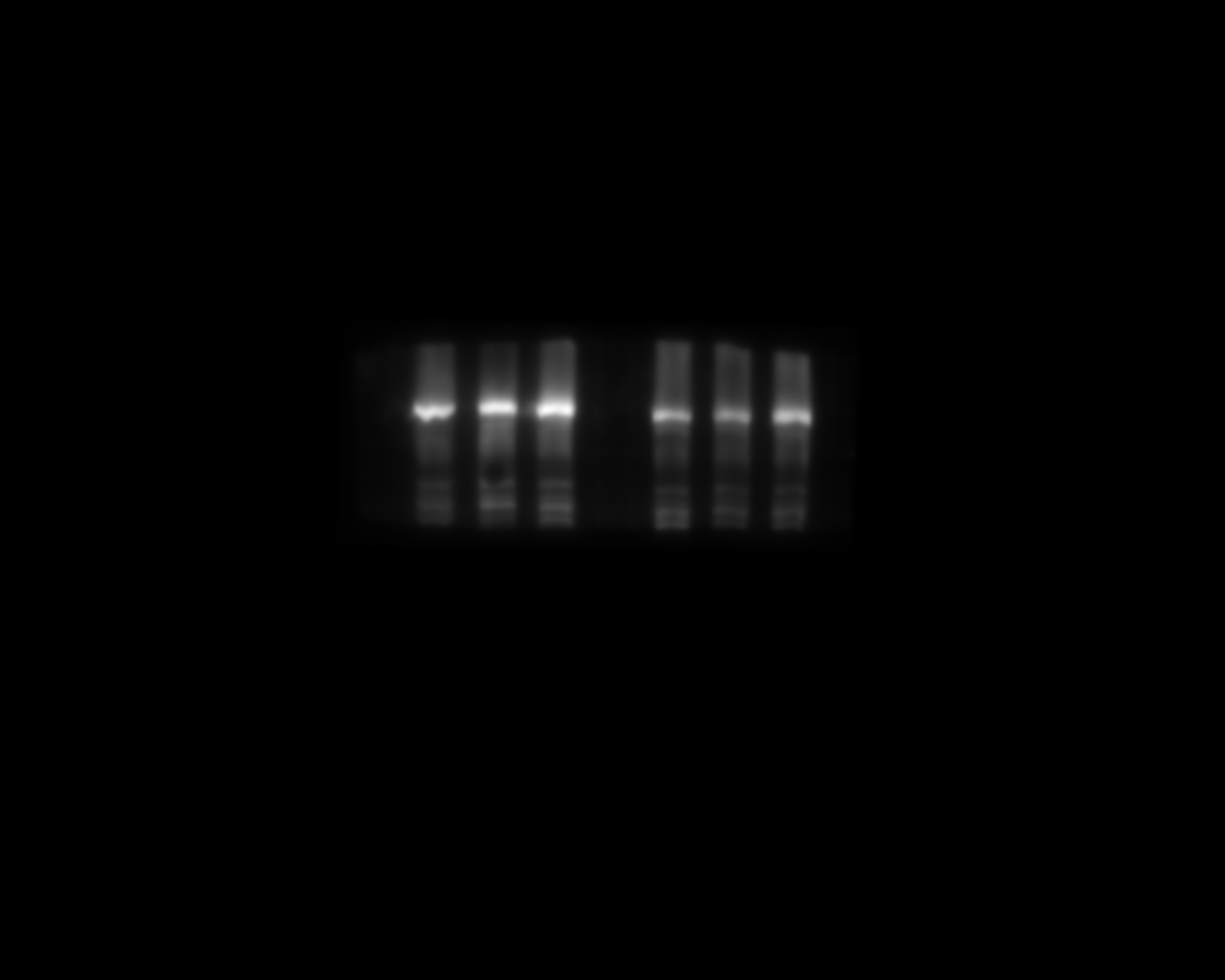

Supplement: Supplementary file 1 [file vetsci-12-00534-s001.zip › Original images/WB/ACACA.tif]

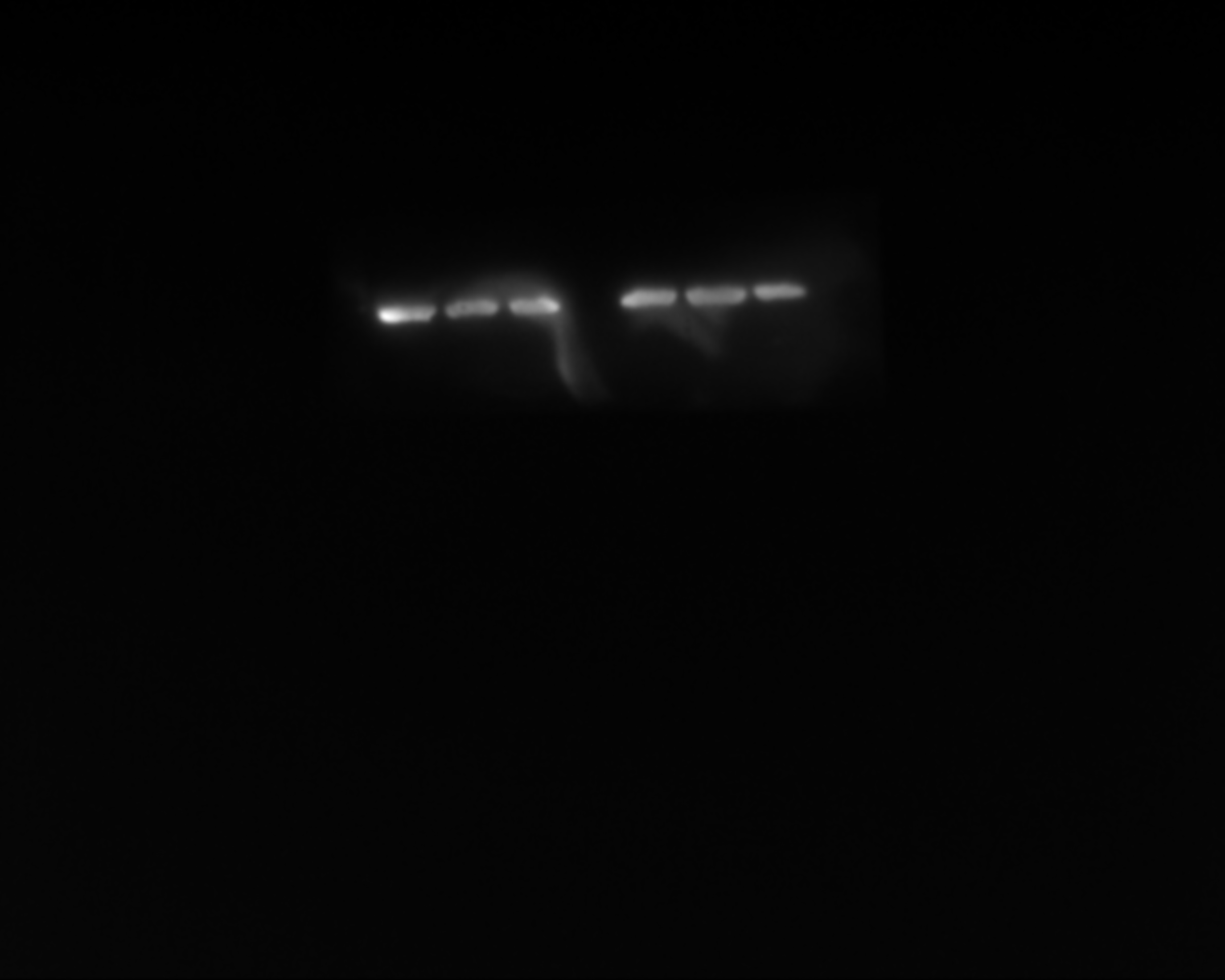

Supplement: Supplementary file 1 [file vetsci-12-00534-s001.zip › Original images/WB/ACTIN.tif]

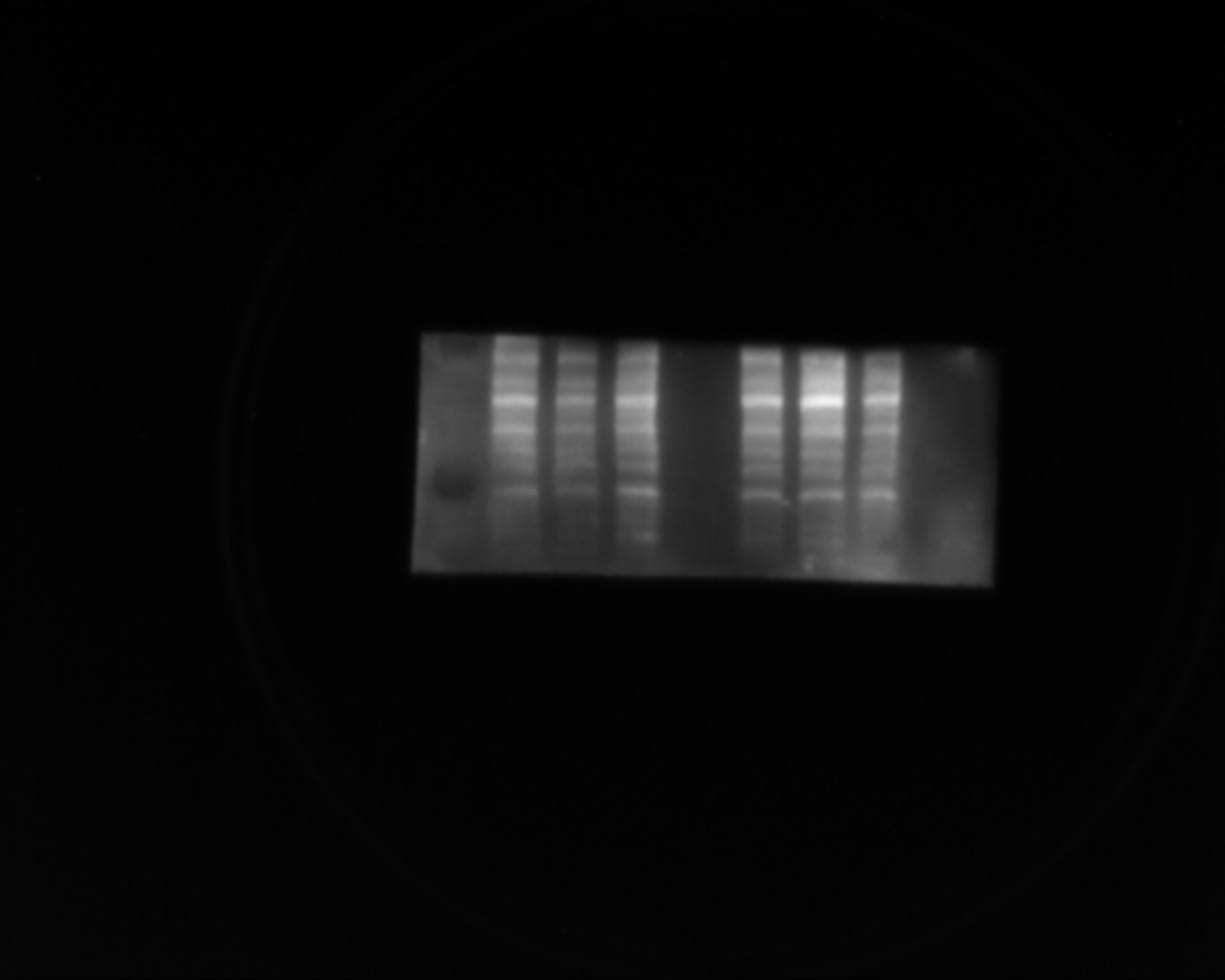

Supplement: Supplementary file 1 [file vetsci-12-00534-s001.zip › Original images/WB/CD36.tif]

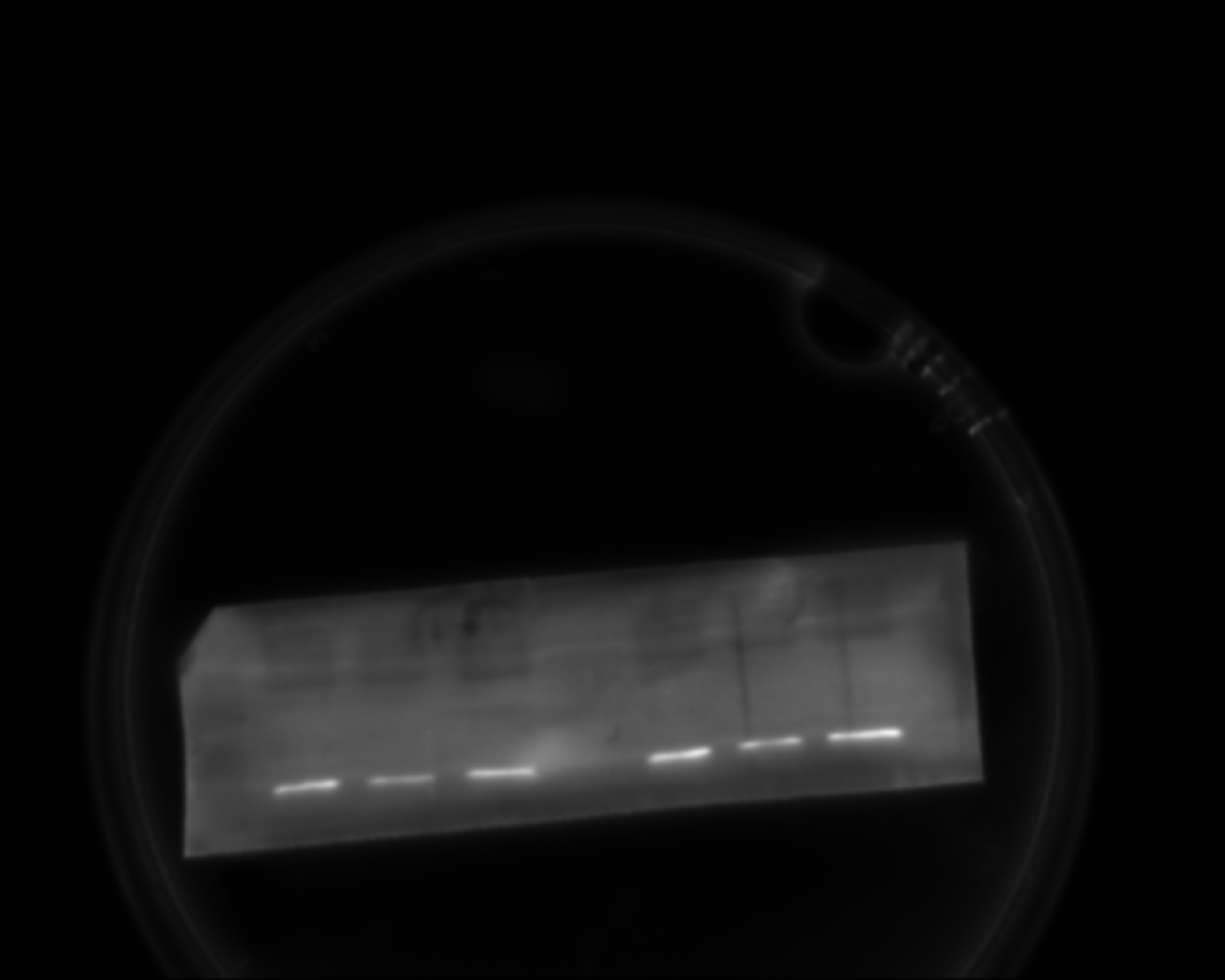

Supplement: Supplementary file 1 [file vetsci-12-00534-s001.zip › Original images/WB/CSN2.tif]

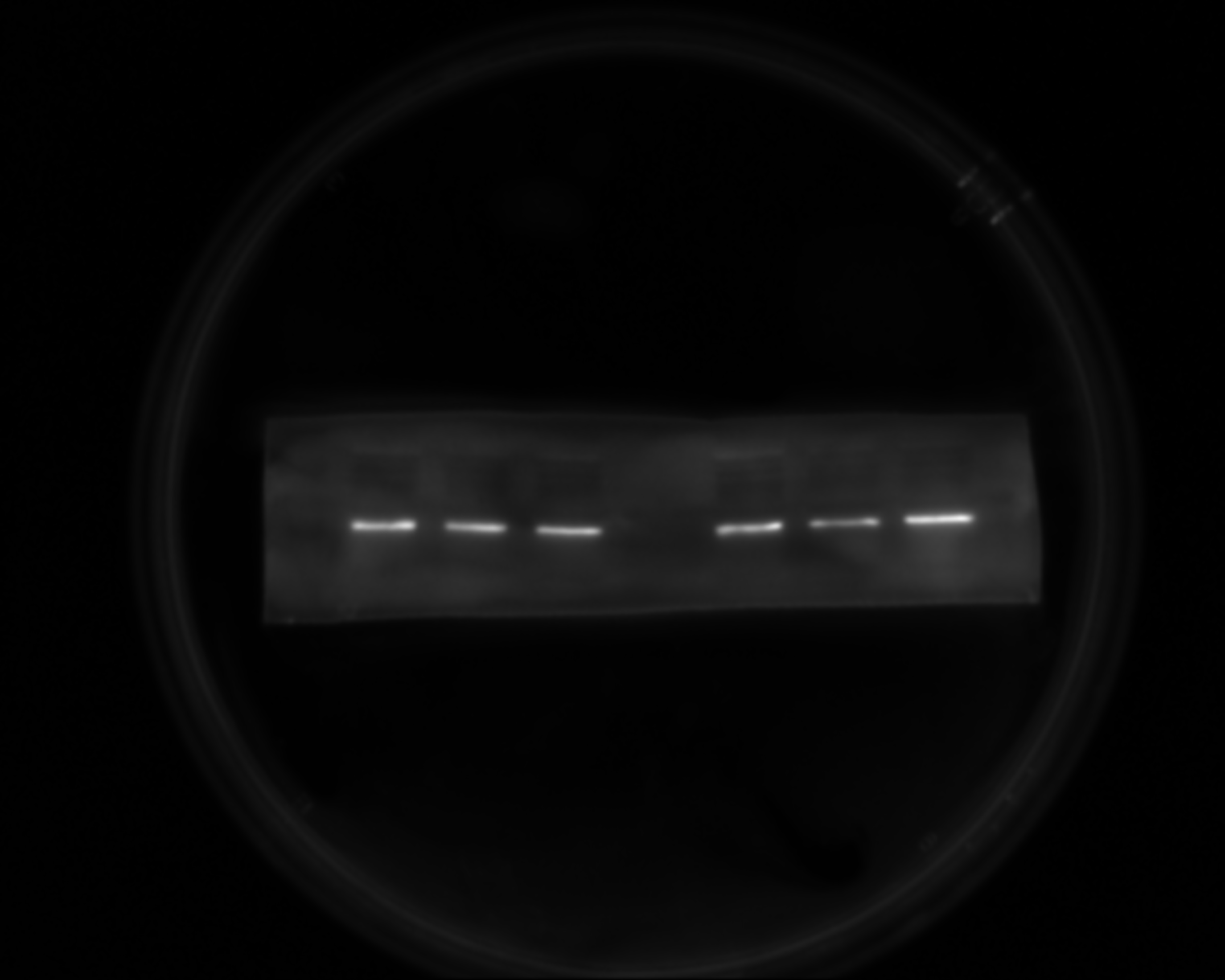

Supplement: Supplementary file 1 [file vetsci-12-00534-s001.zip › Original images/WB/CSN3.tif]

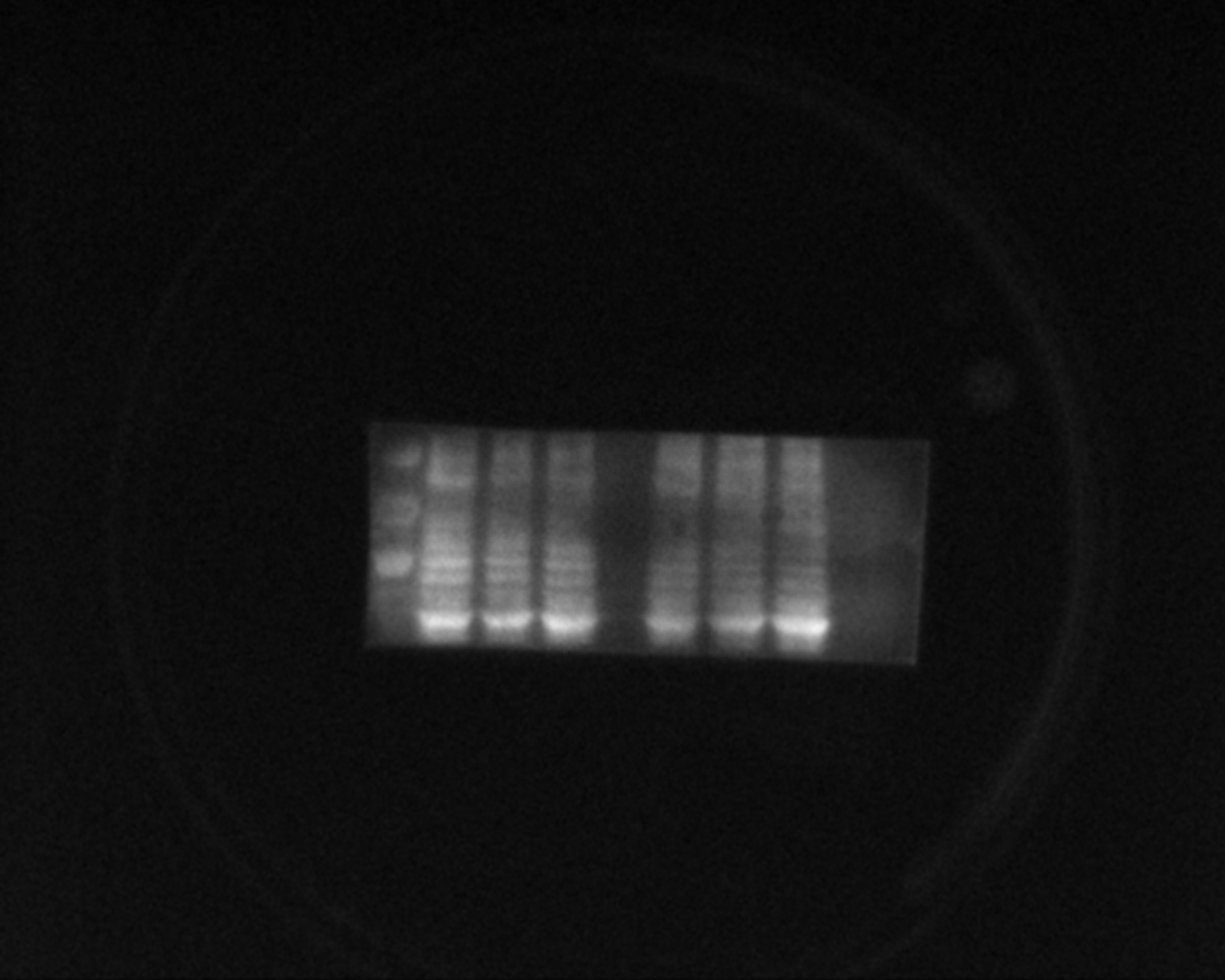

Supplement: Supplementary file 1 [file vetsci-12-00534-s001.zip › Original images/WB/OPN.tif]
